# Genomic Diversity and Footprints of Selection in Hamdani Sheep

**DOI:** 10.3390/biology15181630

**Published:** 2026-09-16

**Authors:** Ebru Demir, Francesco Perini, Eymen Demir, Emiliano Lasagna, Ümit Bilginer, Sarp Kaya, Beste Göneci Fidan, Sezai Alkan, Taki Karslı, Bahar Argun Karslı

**Affiliations:** 1 Department of Agricultural Biotechnology, Faculty of Agriculture, Eskişehir Osmangazi University, Eskişehir 26160, Türkiye; ebrudemr@outlook.com.tr (E.D.); beste.goneci@ogu.edu.tr (B.G.F.); 2 Department of Agricultural, Food and Environmental Sciences, University of Perugia, 06121 Perugia, Italy; francesco.perini@unipg.it (F.P.); emiliano.lasagna@unipg.it (E.L.); 3 Department of Animal Science, Faculty of Agriculture, Akdeniz University, Antalya 07070, Türkiye; eymendemir@akdeniz.edu.tr; 4 Department of Animal and Dairy Sciences, University of Wisconsin, Madison, WI 53705, USA; bilginer@wisc.edu; 5 Department of Medical Services and Techniques, Vocational School of Burdur Health Services, Burdur Mehmet Akif Ersoy University, Burdur 15100, Türkiye; sarpkaya@mehmetakif.edu.tr; 6 Department of Animal Technology, Faculty of Agriculture, Ordu University, Ordu 52200, Türkiye; sezaialkan@odu.edu.tr; 7 Department of Animal Science, Faculty of Agriculture, Eskişehir Osmangazi University, Eskişehir 26160, Türkiye; takikarsli@ogu.edu.tr

**Keywords:** ddRADseq, NGS, molecular genotyping, selection signals, selection signatures

## Abstract

This study aimed to characterize the genomic diversity, population structure, and candidate regions consistent with selection in Hamdani (HAM) sheep using high-density SNP generated by double-digest restriction site-associated DNA sequencing (ddRADseq). A total of 20 animals were sampled, and after quality filtering, 310,663 SNPs from 18 individuals were retained for downstream analyses. Genetic diversity parameters indicated a relatively high level of genomic variation within the breed (*H_O_* = 0.323 ± 0.01 and *H_E_* = 0.326 ± 0.01) and low levels of inbreeding (*F_HOM_* = 0.029 ± 0.02 and *F_ROH_* = 0.008 ± 0.01), suggesting that the population has maintained substantial genetic diversity and has not experienced recent intensive inbreeding. Population structure analyses revealed that HAM sheep form a distinct genetic cluster separated from Turkish fat-tailed breeds, reflecting their unique genetic background and breeding history. Footprints of selection analyses based on runs of homozygosity (ROH) and integrated haplotype score (iHS) identified preliminary candidate genomic regions consistent with selection. After FDR correction, 12 of the 34 trait–chromosome combinations tested for the iHS-derived regions and three of the eight combinations tested for the ROH-derived regions were significantly enriched. Functional annotation highlighted biologically relevant candidate genes involved in reproductive performance (*TSHR*, *FSHR*, and *LHCGR*), lipid metabolism (*ABCG1* and *LPIN1*), and environmental adaptation (*LACC1* and *MBOAT7*). This ddRADseq-based genome-wide characterization provides preliminary information on genetic variability and candidate genomic regions consistent with selection in HAM sheep. Therefore, further studies focusing on a larger sampling strategy across multiple different sheep breeds are needed to design conservation, breeding, and future genomic selection programs.

## Simple Summary

Identification of genetic variability and genomic regions under selection pressure has significant potential not only for sustaining animal production but also for comprehensive breeding programs to address future challenges. As one of the native sheep breeds of Iraq, Hamdani (HAM) sheep are also found in neighboring countries, including Türkiye, owing to their high adaptability to harsh environmental conditions and satisfactory production performance. Therefore, HAM sheep represent a valuable breed for gaining deeper insight into genetic diversity and the effects of natural and artificial selection on the genome. To reduce ascertainment bias associated with SNP-array technologies and capture breed-specific genetic variants, we used double-digest restriction site-associated DNA sequencing (ddRADseq) to investigate genetic diversity and footprints of selection in HAM sheep. The HAM population showed high observed heterozygosity (0.323 ± 0.01) and low genomic inbreeding (mean FHOM = 0.029 ± 0.02; FROH = 0.008 ± 0.01). Selection pressure was identified for numerous candidate genomic regions linked to reproductive performance, lipid metabolism, and environmental adaptation. Given the limited sample size, these findings should be considered preliminary genomic knowledge to support future conservation strategies and genome-based breeding programs for the sustainable improvement of the HAM sheep breed.

## 1. Introduction

Sheep (*Ovis aries*) are among the most economically important livestock species worldwide. Indeed, they not only provide meat, milk, wool, and other products but also play a crucial role in the livelihoods of rural communities, particularly in marginal and semi-arid environments [1,2,3]. Approximately 1400 recognized breeds are currently distributed worldwide, reflecting extensive adaptation to diverse production systems and environmental conditions [4]. In Türkiye, sheep rearing is particularly practiced in semi-arid and mountainous regions by small-scale farming enterprises [5]. Official data reported for 2024 have verified that Türkiye ranks seventh globally with an estimated sheep population of approximately 45 million [3]. Beyond its economic contribution, sheep farming in Türkiye holds significant social and cultural importance, having been an integral part of rural life throughout history. In some regions, traditional nomadic sheep farming practices continue to persist [6].

Possessing a fat-tail phenotype, the Hamdani (HAM) sheep originates from Iraq, while it is geographically distributed in nearby countries, such as Türkiye and Iran [7]. Morphologically characterized by pendulous ears, the breed exhibits high adaptability to arid and semi-arid environments and harsh climatic conditions. HAM sheep are noted for favorable production traits, including strong lamb growth performance, high milk yield, twinning ability, and fleece yield [8,9,10]. Owing to these advantageous productivity and adaptation traits, the HAM breed is among the preferred sheep breeds for breeders in the eastern regions of Türkiye. These characteristics make the breed an important genetic resource for sustainable sheep production under harsh environmental conditions.

Domestication and subsequent breeding have exposed farm animals to both systematic selections based on phenotypic performance and pedigree records, and non-systematic selection driven by natural adaptation or breeder preferences. These processes have generated distinct genomic patterns, known as footprints of selection, which provide critical insights into the evolutionary and breeding history of animal populations [11,12]. The assessment of genetic diversity and footprints of selection is fundamental for designing conservation and breeding strategies, as it provides insights into population history, adaptation, and the genetic basis of economically important traits [12,13]. Genome-wide SNP datasets generated using SNP arrays or next-generation sequencing (NGS) technologies have become indispensable tools for investigating genetic diversity, population structure, and footprints of selection in livestock populations [14]. High-density SNP data generated through NGS-based methods (such as GBS or RADseq) or SNP chips, when combined with advanced statistical and bioinformatic analyses, offer substantial advantages for investigating genetic diversity, phylogenetic relationships, and footprints of selection [4,15]. As SNP chips are designed using reference genomes from specific breeds, they may fail to capture breed-specific genomic variation in local populations. In contrast, ddRADseq targets genome-wide variation without predefined markers, enabling the identification of novel and breed-specific SNPs. Furthermore, the use of two restriction enzymes increases locus coverage, optimizes fragment size selection, and improves SNP detection accuracy while remaining cost-effective for large-scale studies [16]. However, compared with array-based technologies, ddRADseq is more labor- and time-intensive because it involves several consecutive experimental steps. Moreover, its overall genotyping success depends on the correct execution of each step, and reproducibility across runs is not always guaranteed.

Genome-wide SNP data generated using ddRADseq have been widely applied in diverse sheep and other livestock species worldwide to investigate genetic diversity, population structure, phylogenetic relationships [17,18,19], and footprints of selection [20,21,22] through advanced bioinformatic analyses. However, despite the economic importance of the HAM breed, genome-wide characterization based on next-generation sequencing remains unavailable. To date, the most comprehensive genome-wide analysis of the HAM sheep has been conducted using the Illumina Ovine SNP50K BeadChip, generating 53,714 SNPs, and was published only recently [8]. Therefore, this study aimed to characterize the genetic diversity, population structure, and genomic footprints of selection in HAM sheep using high-density genome-wide SNPs generated by ddRADseq. We hypothesized that the higher marker density provided by ddRADseq would enable the identification of previously undetected genomic regions associated with adaptation and economically important traits.

## 2. Materials and Methods

### 2.1. Sampling, DNA Isolation, and Genomic Library Preparation

**Figure 1:**
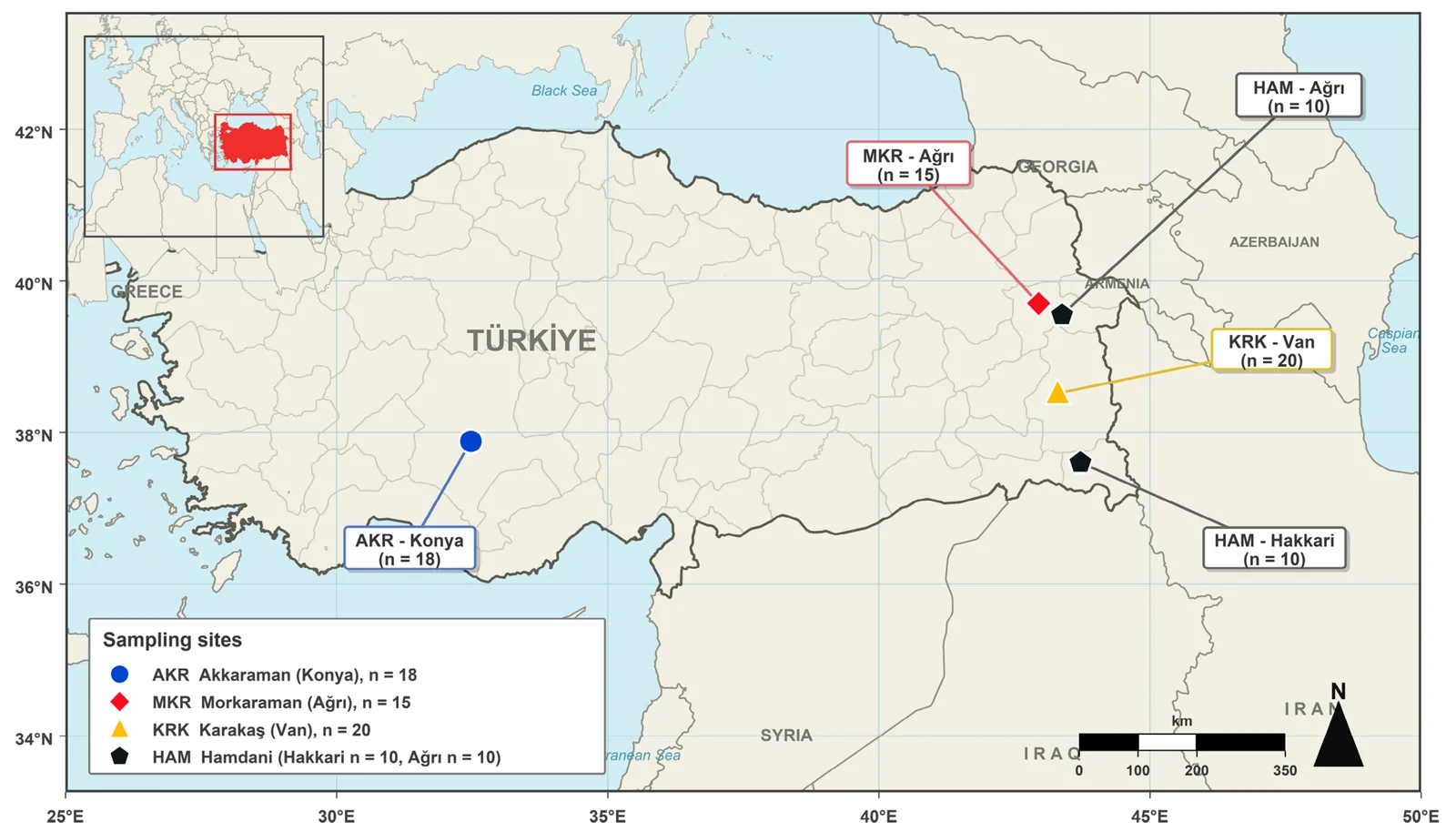
Geographic distribution of sampling sites of the analyzed sheep breeds.

In this study, 20 HAM sheep samples were collected from four different farms located in the provinces of Hakkari and Ağrı (two farms from each province). The sampling locations of the HAM sheep analyzed in this study and the reference breeds (AKR, MKR, and KRK) included from Karsli [23] for the phylogenetic analyses are shown in Figure 1. All sampled animals were females and were selected as unrelated individuals based on pedigree information provided by the breeders. Blood samples were collected by the qualified veterinarians during routine veterinary health management procedures and stored at −20 °C until genomic DNA extraction. Written consent was obtained from the respective farm owners for the use of collected blood samples for scientific research purposes.

Genomic DNA was extracted from 0.5 mL blood samples using the GeneJET Genomic DNA Purification Kit (Thermo K0722, Thermo Fischer Scientific, Waltham, MA, USA). DNA quality and integrity were assessed via 1% agarose gel electrophoresis, and concentrations were determined with the Qubit 4™ Fluorometer (Thermo Fisher Scientific, Waltham, MA, USA) using the DNA HS assay kit (Invitrogen, Waltham, MA, USA). Samples meeting quality and quantity requirements were processed to construct ddRADseq libraries, following the protocol suggested by Peterson et al. [24] with minor modifications adopted by Karsli [23] regarding the selection of the restriction enzyme pair and size selection. DNA was digested with *EcoR*I and *Msp*I restriction enzymes, and the digested products were purified using AmPure XP beads (Beckman Coulter, Brea, CA, USA). Specific adapters and barcodes were ligated to the fragments with T4 DNA ligase, and reverse indexes were assigned to enable pooling. Fragments sized between 300 and 500 bp were PCR-amplified to complete index tagging and enrich the libraries. The final libraries were cleaned and sequenced with paired-end mode on the Illumina NovaSeq 6000 (Illumina, San Diego, CA, USA) platform to generate short reads (2 × 150 bp).

### 2.2. Variant Calling and Data Curation

Short reads were processed using Stacks 2 [25] and assigned to individual samples according to their barcodes. Adapter removal and quality trimming were performed using fastp [26] with default settings. Clean reads were aligned to the ARS-UI_Ramb_v3.0 reference genome using Bowtie2 [27]. Variant calling and filtering were performed using BCFtools [28], applying read-depth (20 ≤ D ≤ 100) and quality (Q ≥ 20) thresholds. Only biallelic SNPs located on autosomes were retained, whereas indels and other variant types were excluded.

SNPs passing BCFtools filtering were subjected to quality control in PLINK [29] using --maf 0.05, --hwe 1e-6, and --geno 0.05. Individuals with more than 10% missing genotypes were removed using --mind 0.1. After filtering, the dataset comprised 18 individuals genotyped for 310,663 SNPs; two samples were excluded because their missing genotype rates exceeded 0.1.

To investigate the genetic relationships of HAM sheep, the present dataset was merged with the ddRADseq dataset reported by Karsli [23], which included 18 Akkaraman (AKR), 15 Morkaraman (MKR), and 20 Karakaş (KRK) sheep. The merged dataset was filtered in PLINK [29] using the same thresholds (--maf 0.05, --hwe 1e-6, --geno 0.05, and --mind 0.1). This procedure yielded 71 individuals genotyped for 278,000 SNPs; the same two HAM samples were excluded because their missing genotype rates exceeded 0.1.

The first dataset, comprising 18 HAM sheep and 310,663 SNPs, was used to assess genetic diversity and identify within-population candidate regions consistent with selection using runs of homozygosity (ROH) and the integrated haplotype score (iHS). The second dataset, comprising 71 individuals from four sheep breeds and 277,194 SNPs, was used for population structure and phylogenetic analyses.

### 2.3. Genetic Diversity Parameters

Genetic diversity indices were estimated using PLINK [29]. Specifically, mean observed heterozygosity (*H_O_*), expected heterozygosity (*H_E_*), and the inbreeding coefficient based on deviations between observed and expected homozygosity (*F_HOM_*) were calculated at the individual and breed levels. In addition, genomic inbreeding based on runs of homozygosity (*F_ROH_*) was derived from the proportion of the autosomal genome encompassed by ROH segments. Breed-level summary statistics, including mean and standard deviation for each parameter in the HAM breed, were computed in R v4.4.1 (https://www.r-project.org, accessed on 20 July 2026).

### 2.4. Genetic Relationships and Population Structure

A Neighbor-Joining (NJ) tree was constructed based on individual allele-sharing distances (1 − IBS) estimated in PLINK [29] and subsequently visualized using FigTree [30]. Bootstrap resampling was not performed; therefore, branch-support values were not available, and the resulting tree topology was interpreted descriptively. Population structure was further investigated using ADMIXTURE [31], testing K values ranging from 2 to 4. The optimal number of ancestral clusters was determined by minimizing the cross-validation error (Supplementary Figure S1). Graphical representation of individual ancestry proportions was generated in R v4.4.1 (https://www.r-project.org, accessed on 20 July 2026) using the BITE package [32].

### 2.5. Detection of Runs of Homozygosity

Runs of homozygosity (ROH) were identified using the detectRUNS package [33] implemented in the R v4.4.1 environment (https://www.r-project.org, accessed on 20 July 2026). ROH were detected using the consecutive-SNP approach, and all subsequent ROH summary statistics and ROH-island analyses were based exclusively on the segments identified using this method. It is noteworthy that no optimized parameters for ROH definition have been validated for the ddRADseq data. Therefore, we selected ROH detection parameters based on conventional thresholds commonly used for ROH detection in genomic datasets with similar SNP densities. To do so, a ROH was required to contain at least 50 consecutive homozygous SNPs, span at least 500 kb, and have a maximum distance of 1 Mb between consecutive SNPs. The minimum length of 500 kb, combined with the requirement for at least 50 consecutive homozygous SNPs, was selected to exclude extremely short homozygous stretches potentially arising by chance or reflecting local linkage disequilibrium, while retaining short ROH potentially informative of older autozygosity [23]. No heterozygous genotypes and a maximum of two missing genotypes were permitted within each ROH. These criteria were applied to reduce the merging of homozygous markers across unsequenced genomic regions. However, the parameters were not specifically calibrated according to the distances between ddRADseq loci, and no sensitivity analysis using alternative thresholds was performed. To identify ROH islands, SNP frequencies within ROH were calculated across individuals. Candidate regions were defined as contiguous SNPs exceeding the 99.9th percentile of the SNP-in-ROH frequency distribution and separated by ≤500 kb. The 99.9th percentile was selected as a stringent empirical threshold retaining the top 0.1% of SNPs most frequently occurring within ROH and was used for exploratory identification of ROH islands rather than as a formal significance threshold [34]. Detected ROH were grouped into five length classes: 0.5–2, 2–4, 4–8, 8–16, and >16 Mb. The lower boundary of the first class reflected the minimum ROH length of 500 kb.

### 2.6. Footprints of Selection Analysis (iHS)

Within-population footprints of selection were investigated in the HAM breed using the integrated haplotype score (iHS) in the rehh package [35] implemented in R software v4.4.1 (https://www.r-project.org, accessed on 20 July 2026). Genotype phasing was performed independently for each autosome using Beagle [36] with default parameters and without an external reference panel.

iHS values were calculated as described by Voight et al. [37] using the scan_hh() and ihh2ihs() functions. The VCF was imported without allele polarization; therefore, no outgroup was used, ancestral and derived allele states were not assigned, and the original REF/ALT coding was retained. Consequently, the sign of iHS was not biologically interpreted, and candidate detection was based on absolute standardized iHS values. Standardization was performed within allele-frequency bins to account for frequency-dependent variation. Extreme markers were defined as those exceeding the empirical 99.9th percentile of the |iHS| distribution, corresponding to |iHS| ≥ 4.279, rather than using a fixed threshold of 4. Because candidate detection was based on an empirical percentile rather than formal *p*-values, no multiple-testing correction was applied to the iHS scan. Candidate regions were identified using non-overlapping 200 kb windows, requiring at least two markers per window and at least two extreme markers. Manhattan plots were generated using the built-in plotting functions of the rehh package [35] in R software v4.4.1 (https://www.r-project.org, accessed on 20 July 2026), with the empirical threshold indicated by horizontal lines.

### 2.7. Functional Annotation and QTL Enrichment

Genes and quantitative trait loci (QTLs) within ±500 kb of ROH islands and iHS candidate regions were identified using the GALLO R package [38]. Gene annotation was based on the *Ovis aries* ARS-UI_Ramb_v3.0 assembly (GTF format), and QTL data were retrieved from the Animal QTLdb (release 58). This fixed interval was used for exploratory positional annotation and was not calibrated using HAM-specific LD decay, as another ovine study suggested [39]. No sensitivity analysis using a narrower annotation window was performed.

QTL enrichment was assessed using the qtl_enrich() function with false discovery rate (FDR) correction for multiple testing. For each trait–chromosome combination, enrichment was evaluated using a hypergeometric test based on the number of QTLs annotated within candidate regions relative to the corresponding number in Animal QTLdb. *p*-values were adjusted using the Benjamini–Hochberg false discovery rate procedure (padj = “fdr”), and adjusted *p* < 0.05 was considered significant. The richness factor was calculated as the ratio between the number of annotated QTLs and the corresponding number in Animal QTLdb. Percentages by QTL category were calculated separately from all marker–QTL overlap records and were not interpreted as enrichment-test statistics.

## 3. Results

### 3.1. Sequencing Quality and Variant Calling

Genomic libraries were constructed from 20 individuals of the HAM sheep breed using the ddRADseq protocol and sequenced on the Illumina NovaSeq 6000 platform. Sequencing generated nearly 371 million paired-end reads with a read length of 150 bp. Approximately 341 million raw reads passed quality filtering and adapter trimming. High-quality reads were successfully aligned to the reference genome, with an average mapping rate of 94.83% (range: 92.72–95.86% across samples). The mean sequencing depth per sample ranged from 21x to 28x, with an overall average depth of 23x. Two different genetic datasets were utilized for downstream analysis, in which genetic variability parameters and footprints of selection in the HAM sheep breed were identified via 310,663 SNP data. The SNP density was consistent with the physical length of autosomes, in which the lowest and highest number of SNPs were identified for chromosome 24 (5102 SNPs) and 1 (25,681 SNPs), respectively. Another dataset covering 277,194 SNPs, on the other hand, was used to uncover the genetic structure of HAM sheep by comparison with three Anatolian sheep breeds, namely AKR, MKR, and KRK [23].

### 3.2. Genomic Diversity and Inbreeding

In the HAM sheep breed, mean *H_O_* and *H_E_* were 0.323 ± 0.01 and 0.326 ± 0.01, respectively. The mean individual inbreeding coefficients were *F_HOM_* = 0.029 ± 0.02 and *F_ROH_* = 0.008 ± 0.01, based on 310,663 SNPs. A total of 404 ROH were detected. Of these, 398 (98.51%) belonged to the 0.5–2 Mb class, with a mean length of 0.850 Mb, whereas six (1.49%) belonged to the 2–4 Mb class, with a mean length of 2.261 Mb. No ROH longer than 4 Mb were observed. Across individuals, the mean number of ROH was 22.44 ± 33.99, with a median of 12 and a range of 2–149 ROH. The mean ROH length per individual was 0.798 ± 0.102 Mb, and the mean total ROH length per individual was 19.56 ± 31.71 Mb.

### 3.3. Population Structure and Divergence

**Figure 2:**
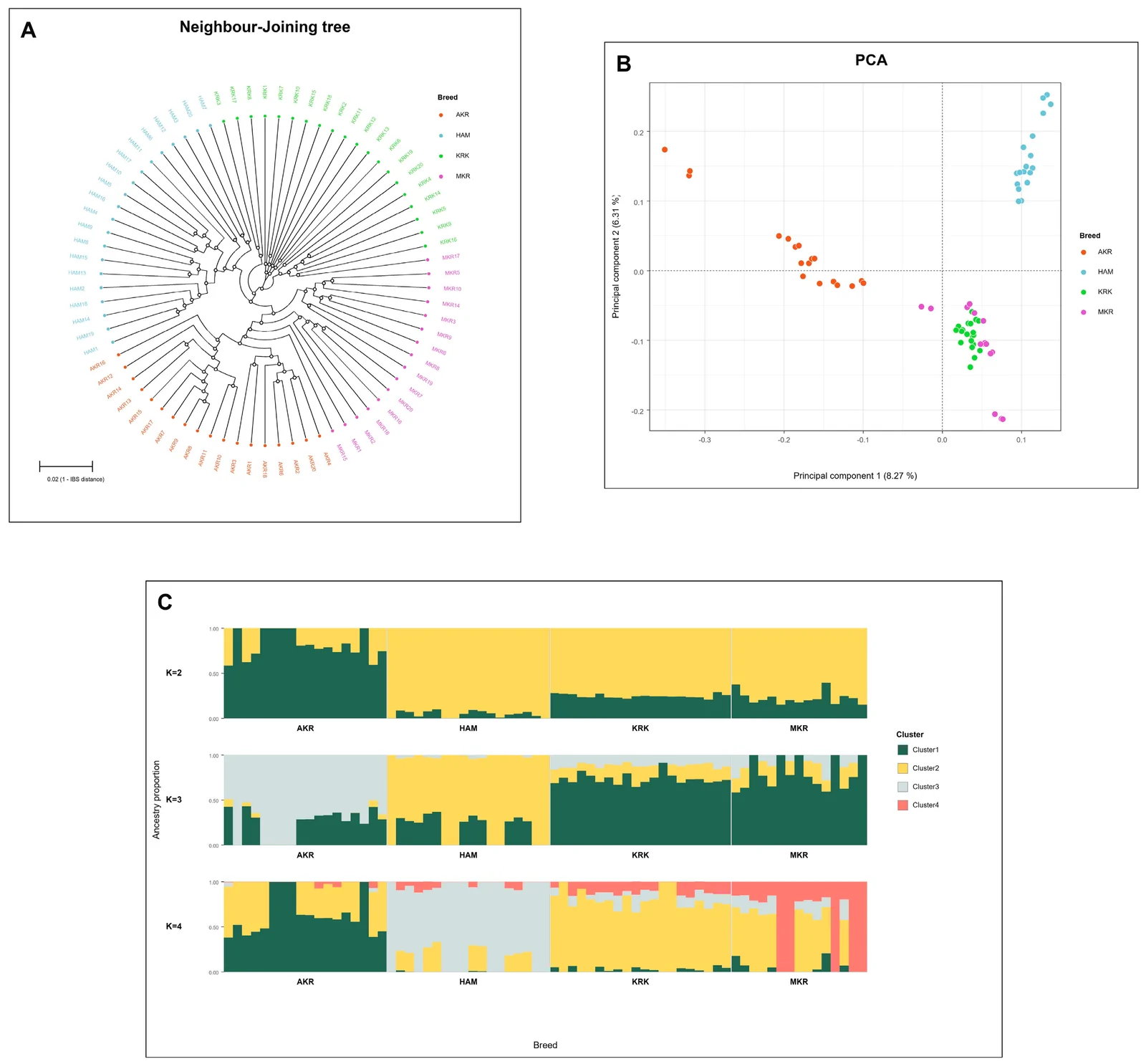
Analyses of population structure and genetic divergence among AKR, HAM, MKR, and KRK breeds. (**A**) Neighbor-joining tree constructed from pairwise individual allele-sharing distances (1 − IBS). (**B**) Principal component analysis (PCA) based on genome-wide SNP data. (**C**) ADMIXTURE ancestry proportions of the 71 analyzed individuals for K values ranging from 2 to 4. Abbreviations: AKR, Akkaraman; HAM, Hamdani; MKR, Morkaraman; KRK, Karakaş.

Based on pairwise individual allele-sharing distances (1 − IBS) estimated from 277,194 autosomal SNPs, the NJ tree grouped individuals from the four breeds into distinct genetic clusters (Figure 2A). Among the 18 retained HAM individuals, mean pairwise IBS was 0.737 ± 0.008 (range: 0.726–0.796; 153 pairwise comparisons), consistent with the pedigree-based selection of unrelated animals. In the PCA, the first two principal components explained 14.58% of the total genetic variance (PC1: 8.27%; PC2: 6.31%), indicating that genetic variation has a multidimensional structure and that within-population differentiation is not strongly captured by a single or a few axes. PCA revealed three major genetic clusters. HAM and AKR formed distinct groups, whereas MKR and KRK clustered closely together (Figure 2B). ADMIXTURE analysis was conducted for K values ranging from 2 to 4, and K = 2 showed the lowest cross-validation error (Supplementary Figure S1). Accordingly, the four breeds were represented by two major ancestry components, as shown in Figure 2C. HAM exhibited a relatively homogeneous genetic profile, whereas KRK and MKR were genetically closer and shared similar ancestral components. In contrast, AKR showed comparatively lower levels of shared ancestry components with the other breeds. Overall, these findings are consistent with the PCA and NJ results, indicating moderate genetic differentiation among breeds alongside a certain degree of shared genetic variation.

### 3.4. Footprints of Selection

**Figure 3:**
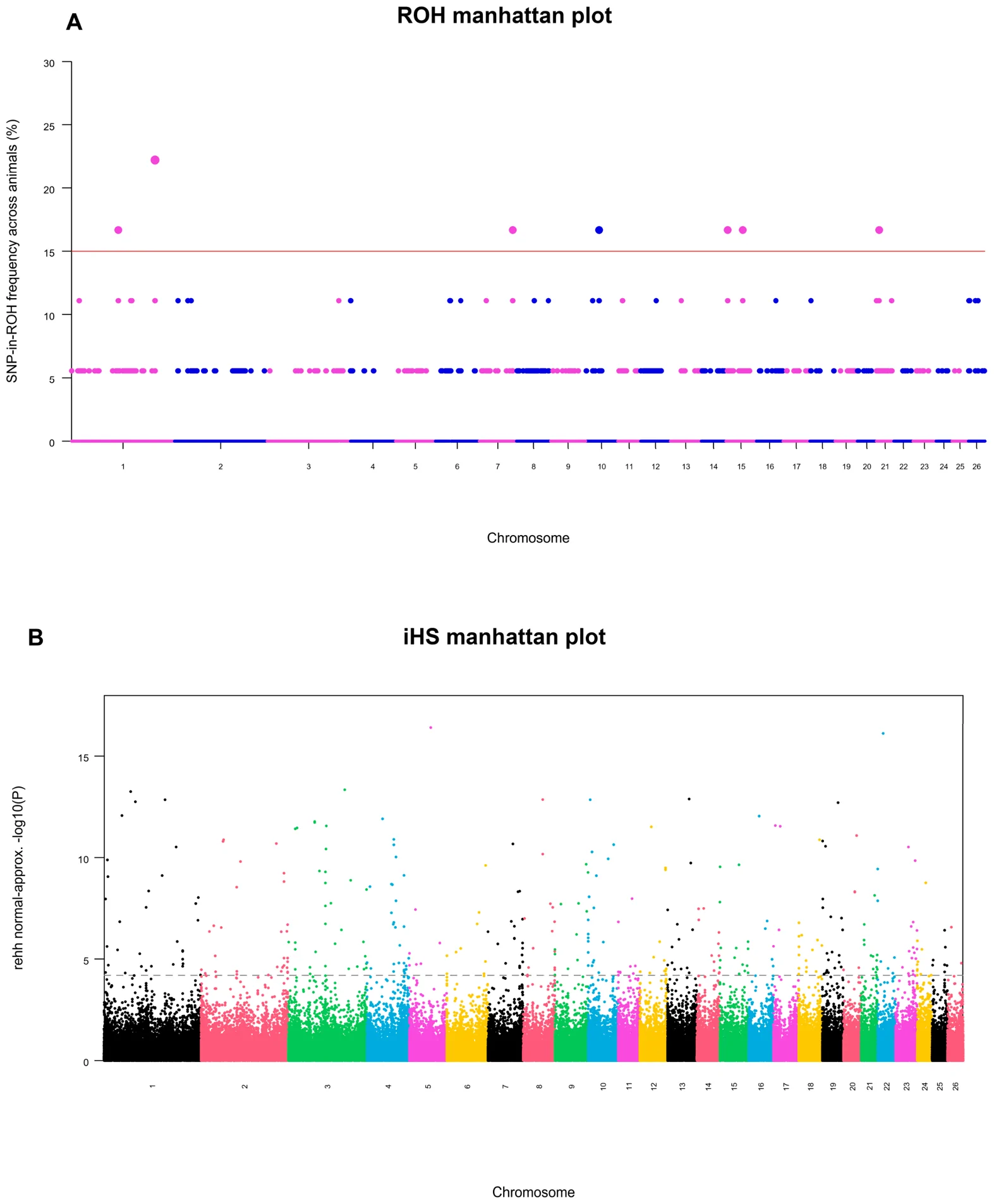
(**A**) Genomic distribution of SNP-in-ROH frequency across HAM animals. Each point represents the percentage of HAM individuals in which a SNP was included within a run of homozygosity. (**B**) iHS selection signals in HAM sheep. Manhattan plot of unpolarized iHS signals. The *y*-axis reports the rehh normal-approximation *p*-value transformed as −log10(P), retained for visualization.

Manhattan plots summarizing the ROH and iHS analyses are presented in Figure 3. The ROH analysis identified seven islands: three on chromosome 1 at 126.05–126.45 Mb (0.41 Mb; 45 SNPs) and 225.56–226.05 Mb (0.49 Mb; 54 SNPs), and; one on chromosome 7 at 90.38–91.12 Mb (0.74 Mb; 63 SNPs); one on chromosome 10 at 36.29–36.64 Mb (0.35 Mb; 15 SNPs); two on chromosome 15 at 4.97–5.49 Mb (0.52 Mb; 48 SNPs) and 45.67–46.11 Mb (0.44 Mb; 44 SNPs); and one on chromosome 21 at 7.14–7.52 Mb (0.38 Mb; 35 SNPs). These islands rest on SNP-in-ROH frequencies corresponding to only three or four of the 18 animals and should therefore be interpreted with caution. Collectively, these islands contained 304 SNPs, while positional annotation within ±500 kb of their boundaries identified 71 distinct genes. The maximum SNP-in-ROH frequency was 16.67%, corresponding to three out of 18 animals for six islands, and 22.22%, corresponding to four animals, for the island located at 225.56–226.05 Mb on chromosome 1. Among the 260,909 SNPs with valid iHS scores, 261 exceeded the empirical 99.9th percentile threshold of ∣iHS∣ ≥ 4.279. The window-based procedure identified 26 candidate genomic intervals containing 64 SNPs, 61 of which exceeded the empirical threshold. Annotation of the iHS candidate regions identified 316 positional candidate genes. Candidate genes identified within iHS and ROH regions are summarized in Supplementary Tables S1 and S2, respectively.

### 3.5. QTL Enrichment and Functional Annotation

**Figure 4:**
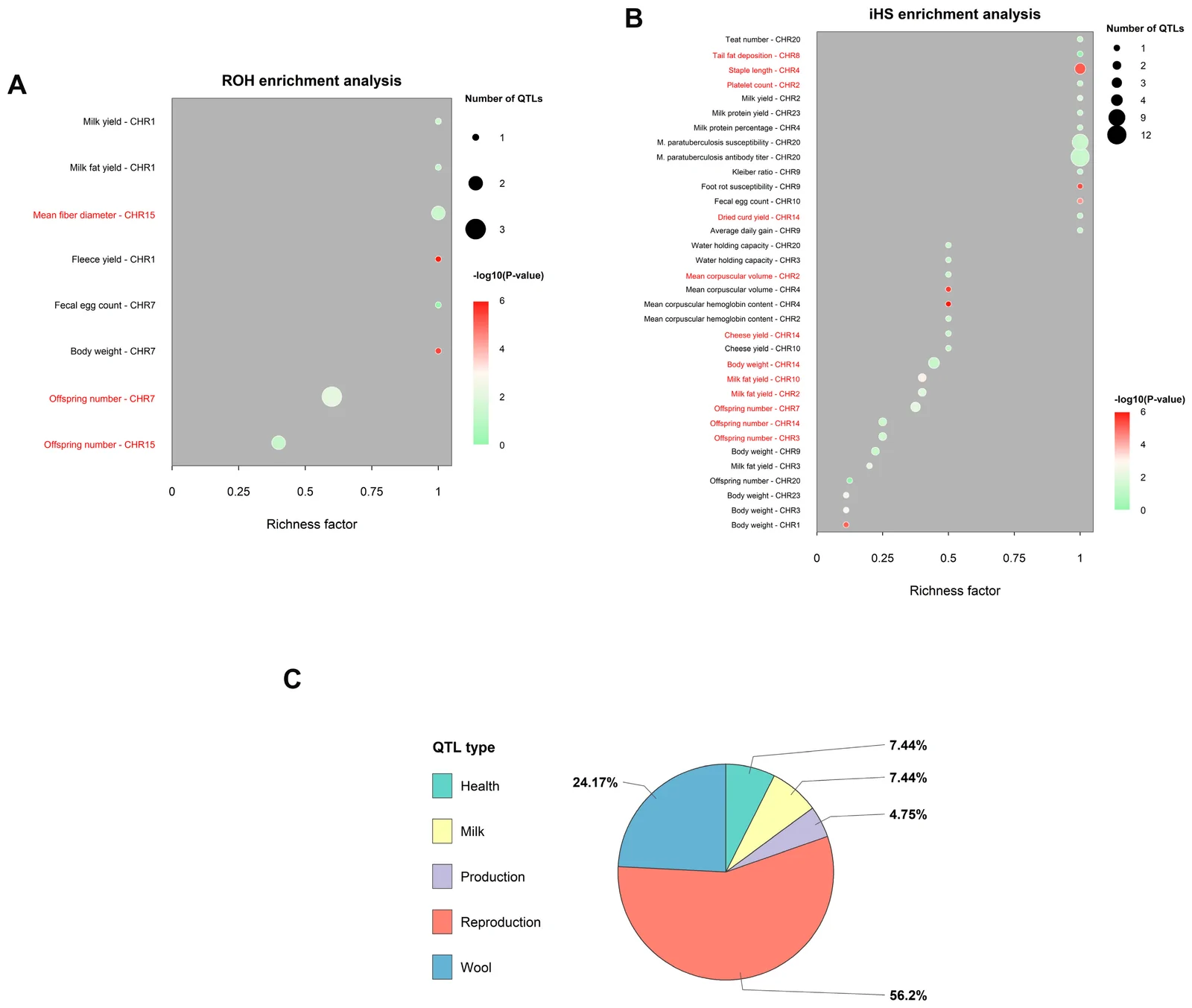
QTL enrichment analyses of candidate genomic regions identified in HAM sheep. (**A**) Enrichment results for ROH-derived candidate regions. (**B**) Enrichment results for iHS-derived candidate regions. In panels A and B, *p*-values that remained significant after Benjamini–Hochberg FDR correction (FDR < 0.05) are highlighted in red. (**C**) Percentage distribution of the 484 marker–QTL overlap records identified for ROH-derived candidate regions according to QTL category.

QTL enrichment analysis of the iHS-derived candidate regions identified 12 significant trait–chromosome combinations among the 34 combinations tested after FDR correction (Figure 4B). These significant combinations, highlighted in red in Figure 4B, involved offspring number on chromosome 3, 7, and 14; body weight, dried curd yield, and cheese yield on chromosome 14; staple length on chromosome 4; tail fat deposition on chromosome 8; milk fat yield on chromosome 2 and 10; and platelet count and mean corpuscular volume on chromosome 2. The remaining 22 combinations did not survive FDR correction and were therefore not interpreted as enriched. Independently of the enrichment test, the 141 marker–QTL overlap records comprised 62 health-related records (43.97%), 25 milk-related records (17.73%), 22 production-related records (15.60%), 16 reproduction-related records (11.35%), eight wool-related records (5.67%), six meat-and-carcass records (4.26%), and two exterior-trait records (1.42%). Complete results for the iHS-derived candidate regions, positional genes, marker–QTL overlaps, and QTL enrichment analysis are provided in Supplementary Table S1.

QTL enrichment analysis of the ROH-derived candidate regions identified three significant trait–chromosome combinations among the eight combinations tested after FDR correction (Figure 4A). The significant combinations, highlighted in red in Figure 4A, comprised offspring number on chromosome 7 and chromosome 15 and mean fiber diameter on chromosome 15. The remaining five combinations—fleece yield, milk yield, and milk fat yield on chromosome 1 and fecal egg count and body weight on chromosome 7—did not survive FDR correction and were therefore not interpreted as enriched. Independently of the enrichment test, 272 of the 484 marker–QTL overlap records were related to reproduction (56.20%), 117 to wool traits (24.17%), 36 to health (7.44%), 36 to milk traits (7.44%), and 23 to production traits (4.75%; Figure 4C). Complete results for the ROH islands, positional genes, marker–QTL overlaps, and QTL enrichment analysis are provided in Supplementary Table S2.

A literature survey revealed that the candidate genes identified within ROH regions under selection pressure in HAM sheep have previously been associated with a wide range of phenotypic traits, including body conformation, growth, pigmentation, reproductive performance, and environmental adaptation (Table 1). In particular, *STON2*, *SEL1L*, *GJB2*, *GJA3*, and *MRPL17* have been associated with body weight and conformation traits, whereas *RAB38* has been linked to pigmentation-related characteristics. Furthermore, *TSHR*, *GTF2A1*, *CEP128*, *GJB6*, *MMP13*, and *DYNC2H1* have been reported to influence reproductive traits across different livestock species. *TSHR*, *ZMYM2*, *PSPC1*, *GJB6*, *GJB2*, and *GJA3* genes, on the other hand, have been linked to environmental adaptation and stress tolerance. Consistent with previous studies in livestock species, these findings suggest that the ROH regions identified in HAM sheep harbor footprints of selection associated with morphological characteristics, productive performance, and adaptation to local environmental conditions.

## 4. Discussion

Relying on 310,663 SNP data across the genome, this study has confirmed that HAM sheep conserve a high genome-wide variability (*H_O_* = 0.323 ± 0.01 and *H_E_* = 0.326 ± 0.01) together with a low inbreeding level (*F_HOM_* = 0.029 ± 0.02). Consistent with the low genomic inbreeding coefficient, the *F_ROH_* (0.008 ± 0.01) estimate further supports limited autozygosity within the HAM population. These findings are in agreement with previous genome-wide studies that reported moderate to high levels of genetic diversity in HAM sheep populations reared in Türkiye [78].

Utilizing 396,097 SNPs recovered by the ddRADseq technique, Karsli [23] reported observed heterozygosity values ranging from 0.296 to 0.315 and expected heterozygosity values varying between 0.287 and 0.299 for the Akkaraman, Güney Karaman, Morkaraman, and Karakas breeds. HAM sheep showed slightly higher heterozygosity values compared to the values reported for the Akkaraman, Güney Karaman, Morkaraman, and Karakas breeds [23]. This difference may be attributable to variations in the demographic histories, effective population sizes, selection pressures, and breeding systems of the populations examined. The ongoing animal movements in borderlands may have contributed to the maintenance of genome-wide variability in HAM sheep. Similarly, low inbreeding values in terms of *F_HOM_* and *F_ROH_*, both of which were close to zero, further support the lack of substantial heterozygosity loss within the population. Moreover, the marked predominance of short ROH (0.5–2 Mb) is consistent with background autozygosity inherited from relatively distant common ancestors, because recombination progressively fragments ancestral haplotypes across generations [79]. Taken together, these findings indicate that the HAM sheep breed maintains sufficient genomic variability, which could be utilized for future breeding and conservation programs.

ADMIXTURE analysis revealed two ancestral populations, while NJ tree and PCA analyses have separated four sheep breeds (HAM, AKR, MKR, and KRK) into three genetic clades, consistent with their breeding practices, geographical distributions, and historical backgrounds. HAM and AKR breeds were clearly separated, while MKR and KRK were clustered together. In particular, the formation of a separate cluster by HAM individuals in the NJ tree, together with their clear separation from the other breeds along the second principal component in the PCA, indicates that this breed has retained a distinct genetic background. In contrast, the close clustering of MKR and KRK individuals in NJ tree and PCA analyses demonstrates a close genetic relationship between them. This observation is further supported by the ADMIXTURE analysis, in which MKR and KRK individuals shared similar ancestral genetic components. These findings are consistent with previous reports showing that the KRK and MKR breeds, both reared in eastern Türkiye, share a common genetic background [80,81]. Despite its geographical proximity to KRK and MKR, HAM sheep formed a genetically distinct cluster, suggesting a partially independent breeding history and limited recent admixture with the other indigenous populations. This finding indicates that historical geographic isolation and selection practices for diverse purposes have the potential to differently shape the genome of various sheep populations raised in a certain geographic zone. Nevertheless, the genetic affinity of HAM sheep with Turkish indigenous breeds should be interpreted within the broader historical context of sheep populations in the Anatolian–Fertile Crescent region. Long-standing pastoral movements, transhumance practices, and livestock exchanges across present-day political boundaries may have facilitated the sharing of ancestral genetic components among neighboring populations. Thus, the current genetic structure of HAM sheep may represent the combined effects of shared historical ancestry, subsequent breed-specific differentiation driven by local management practices, and environmental adaptation.

In the present study, the integrated analyses of iHS and ROH revealed genomic regions harboring footprints of selection associated with economically and adaptively important traits in HAM sheep. This interpretation is supported by the complementary properties of the two approaches. Indeed, iHS is particularly sensitive to ongoing or recent positive selection acting on alleles that have not yet reached fixation, whereas ROH segments represent regions of extended homozygosity, reflecting the cumulative effects of historical selection, demographic process, and inbreeding [37,82]. The results indicate that the enrichment of QTLs related to reproduction, growth, milk production, wool characteristics, health, and adaptation reflects the contribution of both recent and historical selection pressures to the shaping of the current genomic architecture of this breed. Similar patterns have been reported in indigenous sheep populations, where both natural and artificial selection jointly influence genomic regions associated with productivity and environmental adaptation [8,18,83]. The observed enrichment of QTLs associated with reproductive, growth, milk, and wool traits, as well as adaptive characteristics, suggests that the HAM genome has been shaped by a combination of natural and artificial selection pressures linked to local production systems and environmental conditions.

The iHS analysis identified 26 genomic intervals associated with 141 marker–QTL overlap records. Of these records, 62 were related to health (43.97%), 25 to milk traits (17.73%), and 22 to production traits (15.60%). These descriptive percentages were calculated from all marker–QTL overlap records and should not be interpreted as enrichment statistics. After FDR correction, 12 of the 34 tested trait–chromosome combinations remained significantly enriched. The presence of numerous QTLs within the detected selection regions, including those associated with susceptibility to *Mycobacterium paratuberculosis* and antibody titers, body weight, litter size, milk fat yield, and wool traits, indicates that this indigenous breed has adapted to multi-purpose production systems. Similarly, previous studies in HAM and other sheep breeds have consistently reported that footprints of selection are frequently enriched in genomic regions associated with growth, reproduction, immune response, and milk production traits [4,8,84]. Collectively, these findings indicate that the identified footprints of selection in HAM sheep are biologically meaningful and reflect the measurable impact of historical breeding practices on the genome.

All iHS-derived candidate genes discussed herein, together with their corresponding chromosomes and genomic positions, are provided in Supplementary Table S1. Among the candidate genes identified within the detected selection regions, *ABCG1*, *PRDM15*, *PDE9A*, *SLC37A1*, *UBASH3A*, and *RCC2* are of particular interest. Notably, the *ABCG1* gene is involved in lipid transport and metabolism [85] and may therefore be associated with selection processes related to milk fat yield and energy metabolism. In addition, *PRDM15*, a transcriptional regulator involved in embryonic development and the control of cell differentiation [86], may play a role in selection pressures linked to reproductive performance and developmental traits. *PDE9A*, which is involved in the regulation of the cGMP signaling pathway [87], is known to influence a range of physiological processes. Taken together, these findings suggest that the footprints of selection detected in HAM sheep reflect not only production-related performance but also complex biological mechanisms underlying environmental adaptation, immune response, and reproductive success. Therefore, these genomic regions may represent important candidate loci for future functional genomics studies and genomic selection programs.

The iHS analysis indicated that recent positive selection in HAM sheep has acted on multiple genes associated with reproduction, growth, metabolism, and environmental adaptation. In particular, the *FSHR* and *LHCGR* genes, which play critical roles in ovarian follicular development, ovulation, and fertility [88], showed notable selection signals, suggesting selection pressures aimed at improving reproductive performance. The *TSHR* gene, identified in both iHS and ROH analyses, has been linked to reproductive seasonality, metabolic regulation, and environmental adaptation, indicating a potential role in the adaptive traits of HAM sheep. Furthermore, footprints of selection detected in lipid-metabolism-related genes such as *ABCG1* and *LPIN1* [89,90], as well as genes involved in growth and energy metabolism including *JAZF1*, *FOXN3*, *GREB1*, *STEAP2*, and *ROCK2* [91,92,93,94], support selection processes related to body weight, fat deposition, and overall production performance. In addition, the identification of immune- and stress-related genes such as *LACC1* and *MBOAT7* [95,96] suggests that health and environmental resilience have also been under selective pressure. Overall, these findings demonstrate that both historical and recent selection pressures have played a significant role in shaping genomic regions associated with reproductive success, energy utilization, growth performance, immune capacity, and environmental adaptation in HAM sheep.

Among the 484 marker–QTL overlap records identified for the ROH-derived regions, reproduction and wool represented the most frequent descriptive categories. However, formal enrichment testing supported only offspring number on chromosome 7 and 15 and mean fiber diameter on chromosome 15 after FDR correction (Supplementary Table S2). Reproductive performance is one of the primary determinants of economic efficiency in sheep production systems, particularly under extensive management conditions. Previous studies have shown that footprints of selection in indigenous sheep populations are frequently enriched for genes associated with fertility and reproductive success, reflecting the long-term importance of these traits for population sustainability [97,98]. In the present study, several genes identified within ROH regions, including *TSHR*, *GTF2A1*, *CEP128*, *GJB6*, *DYNC2H1*, and *MMP13*, have previously been associated with litter size, fertility, and other reproductive traits across different animal species (Table 1). However, because the ROH-island signals were supported by SNP-level sharing in only three to four of the 18 animals, they may partly reflect individual autozygosity or sampling variation and should therefore be interpreted cautiously. Among these candidate genes, *TSHR* appears particularly noteworthy. Beyond its established role in regulating thyroid hormone signaling, *TSHR* has repeatedly been reported as a target of selection in domesticated animals and has been associated with reproductive performance, seasonality, and environmental adaptation in sheep and other livestock species [47,48]. The identification of *TSHR* within ROH regions may therefore reflect dual selection pressures related to both reproductive efficiency and adaptation to the harsh climatic conditions characteristic of the regions where HAM sheep are traditionally raised.

Although high-density genetic data covering 310,663 SNPs were utilized to perform genetic variability and footprint of selection analyses, this study has focused on a single breed with a low number of samples. The limited sample size of 18 HAM individuals reduces the statistical power and the representativeness of the results at the breed level, particularly for population-level ROH islands and extreme iHS signals. Moreover, iHS was calculated from only 36 statistically inferred haplotypes phased without an external reference panel, and phasing accuracy was not independently evaluated; therefore, phase uncertainty may have affected the iHH estimates and resulting iHS signals. Therefore, the identified genomic regions should be regarded as preliminary candidates consistent with selection and require validation in a larger and independently sampled HAM population. Alternatively, reference-based phasing may be utilized for further studies carried out with low sample size. Given a lack of reference-based phasing, the findings of this study should be considered preliminary results, and the biological interpretation of candidate genes identified in this study should be considered with caution. Several genes have been associated with economically important traits in livestock species, while evidence obtained from cattle, pigs, chickens, or other organisms cannot be directly extrapolated to sheep without further validation. Therefore, these genes should be considered as potential candidates rather than confirmed causal genes. Given the limitations of this paper (low sample size and lack of post-validation efforts), further studies with more breeds and sample sizes are encouraged to not only validate the current findings but also to identify the effect of genomic regions under selection pressure on phenotypic traits by comparison of sheep breeds reared for different purposes.

## 5. Conclusions

This study provides a ddRADseq-based assessment of genetic diversity, population structure, and candidate genomic regions consistent with selection in HAM sheep. The results indicated relatively high genetic diversity, low genomic inbreeding, and a distinct genetic profile compared with the indigenous Turkish sheep breeds included in the analysis. In particular, the ROH islands were supported by SNP-in-ROH frequencies corresponding to only three to four animals and may therefore be sensitive to sampling variation. After FDR correction, QTL enrichment analysis supported 12 of the 34 trait–chromosome combinations tested for the iHS-derived regions and three of the eight combinations tested for the ROH-derived regions. The significant ROH-derived combinations were restricted to offspring number on OAR7 and OAR15 and mean fiber diameter on OAR15. Positional candidate genes within the identified regions have previously been associated with reproductive performance, lipid metabolism, and environmental adaptation; however, these literature-based associations do not demonstrate causal relationships with the corresponding traits. Overall, the findings provide preliminary genomic information that may contribute to the characterization and conservation of HAM sheep. Nevertheless, the limited sample size and uncertainty associated with haplotype phasing without an external reference panel require validation of these results in larger and independently sampled populations before their application in conservation or breeding programs.

## Figures and Tables

**Table 1:** Candidate genes identified under selection pressure in HAM sheep via ROH analysis and their possible phenotypic effects.

No.	Gene	Chr	Effect(s) on Phenotype/Species	References
1	*CLDN8*	1	Somatic cell score (Cattle), Horn development (Cattle)	Wolf et al. [40]; Campos et al. [41]
2	*CLDN17*	1	Birth weight (Sheep), Reproductive traits (Pig)	Abousoliman et al. [42]; Zhang et al. [43]
3	*CEP128*	7	Prolificacy (Sheep) Growth and development (Pig)	Qiao et al. [44]; Ding et al. [45]
4	*TSHR*	7	Reproductive traits (Sheep) Litter size (Sheep) Adaptation to life in warmth (Sheep) Cold climate adaptation (Cattle) Egg production (Chicken)	Zhao et al. [46]; Tao et al. [47]; Jin et al. [48]; Wu et al. [49]; Zhang et al. [50]
5	*GTF2A1*	7	Reproductive traits (Sheep) Litter size (Sheep) Egg production (Chicken)	Zhao et al. [46]; Hamadalahmad et al. [51]; Zhang et al. [50]
6	*STON2*	7	Body weight and conformation (Goat) Egg production (Chicken)	Selionova et al. [52]; Zhang et al. [50]
7	*SEL1L*	7	Body weight and conformation (Goat) Milk fat percentage (Cattle) Reproductive traits (Sheep)	Selionova et al. [52]; Abdulrahman et al. [53]; Ospina et al. [54]
8	*GJB6*	10	Reproductive traits (Sheep) Transport activity, hearing functions and ear development (Goat) Adaptation to environment (Goat)	Li et al. [55]; Tsartsianidou et al. [56]; Pegolo et al. [57]
9	*GJB2*	10	Body size and growth (Goat) Transport activity hearing functions and ear development (Goat) Adaptation to environment (Goat)	Onzima et al. [58]; Tsartsianidou et al. [56]; Pegolo et al. [57]
10	*GJA3*	10	Body size and growth (Goat) Adaptation to environment (Goat)	Onzima et al. [58]; Pegolo et al. [57]
11	*ZMYM2*	10	Adaptation to heat stress (Goat) Adaptive functions (Goat)	Fernandes Júnior et al. [59]; Deniskova et al. [60]
12	*PSPC1*	10	Fertility traits (Beef cattle, Review) Environmental adaptation (Goat)	Colombi, et al. [61]; Peng et al. [62]
13	*MPHOSPH8*	10	Marbling traits (Cattle) Breast weight (Chicken) Adaptive functions (Goat)	Su et al. [63]; Kanlisi et al. [64]; Deniskova et al. [60]
14	*PARP4*	10	Carcass weight (Cattle) Breast weight (Chicken) Adaptive functions (Goat)	Edea et al. [65]; Kanlisi et al. [64]; Deniskova et al. [60]
15	*DYNC2H1*	15	Litter size (Pig) Fiber diameter (Sheep) Milk yield (Cattle)	Ding et al. [66]; Ramos et al. [67]; Vani et al. [68]
16	*DCUN1D5*	15	Age at first egg (Chicken)	Kudinov et al. [69]
17	*MMP13*	15	Litter size (Pig) Meat quality (Sheep) Somatic cell score (Cattle),	Ding et al. [45]; Zhao et al. [70]; Chernet et al. [71];
18	*MMP12*	15	Meat quality (Sheep) Somatic cell score (Cattle),	Zhao et al. [70]; Chernet et al. [71]
19	*MMP1*	15	Somatic cell score (Cattle),	Chernet et al. [71]
20	*MRPL17*	15	Body conformation trait (Horse)	Shen et al. [72]
21	*DCHS1*	15	Milk yield (Cattle)	Pedrosa et al. [73]; Saravanan et al. [74]
22	*RAB38*	21	Pigmentation (Cattle) Plumage color (Chicken) Coat color (Alpaca)	Hollmann et al. [75]; Mastrangelo et al. [76]; Melo-Rojas et al. [77]

## Data Availability

The data (including raw reads and reference-aligned SNP data) presented in this study are available upon request from the corresponding author.

## Supplementary Materials

The following supporting information can be downloaded at: https://www.mdpi.com/article/10.3390/biology15181630/s1, Table S1: iHS-derived candidate regions, positional candidate genes, marker–QTL overlaps, and QTL enrichment statistics; Table S2: ROH islands, positional candidate genes, marker–QTL overlaps, and QTL enrichment statistics. Figure S1: ADMIXTURE cross-validation error for K = 2–6.

## Abbreviations

The following abbreviations are used in this manuscript:

AKR	Akkaraman
ddRADseq	Double-digest restriction site-associated DNA sequencing
GBS	Genotyping-by-sequencing
HAM	Hamdani
Ihh	Integrated extended haplotype homozygosity
iHS	Integrated haplotype score
KRK	Karakaş
MKR	Morkaraman
NGS	Next-generation sequencing
PCA	Principal component analysis
RADseq	Restriction site-associated DNA sequencing
ROH	Runs of homozygosity
SNP	Single nucleotide polymorphism

## References

[1] Zeder M.A. (2008). Domestication and early agriculture in the Mediterranean basin: Origins, diffusion, and impact. Proc. Natl. Acad. Sci. USA.

[2] Larson G., Fuller D.Q. (2014). The evolution of animal domestication. Annu. Rev. Ecol. Evol. Syst..

[3] FAO (2024). FAOSTAT, Food and Agriculture Organization of the United Nations, Food and Agriculture Data. https://www.fao.org/faostat/en/#data/QCL.

[4] Argun Karsli B., Demir E., Bilginer U., Dogru H., Karsli T., Kaya S. (2024). Genome-wide discovery of selection signatures in four Anatolian sheep breeds revealed by ddRADseq. Sci. Rep..

[5] Aydin K.B., Bi Y., Brito L.F., Ulutaş Z., Morota G. (2024). Review of sheep breeding and genetic research in Türkiye. Front. Genet..

[6] Ayan S., Yılmaz A. (2024). A Study on the Technical and Structural Characteristics of Nomadic Sheep Breeding Enterprises: Diyarbakır Province Example. J. Anim. Prod..

[7] Bingöl E., Bingöl M. (2018). Some growth, reproduction and lactation characteristics of hamdani sheep. Yuz. Yil Univ. J. Agric. Sci..

[8] Bayraktar M., Shoshin O., Saravanan K.A., Al-Thuwaini T.M., Gökçe G., Fadhil Z.H., Güney M.Ç. (2025). Analysis of Population Structure and Selection Signatures in Two Iraqi Fat-Tailed Sheep Breeds. Vet. Med. Sci..

[9] Alkass J.E., Mustafa K.N., Hidayet H.M., Baker I.A. (2025). Performance of Hamdani Sheep in Iraq: A Review. Zanin J. Sci. Eng..

[10] Aljubouri T.R., Al-Khafaji F.M., Al-Shuhaib M.B.S. (2025). Comparative Analysis of Sheep Growth Traits in Karakul, Al-Naimi, and Hamdani Breeds. Assiut Vet. Med. J..

[11] Gouveia J.J.D.S., Silva M.V.G.B.D., Paiva S.R., Oliveira S.M.P.D. (2014). Identification of selection signatures in livestock species. Genet. Mol. Biol..

[12] Saravanan K.A., Panigrahi M., Kumar H., Bhushan B., Dutt T., Mishra B.P. (2020). Selection signatures in livestock genome: A review of concepts, approaches and applications. Livest. Sci..

[13] Groeneveld L.F., Lenstra J.A., Eding H., Toro M.A., Scherf B., Pilling D., Negrini R., Finlay E.K., Jianlin H., Groeneveld E. (2010). Genetic diversity in farm animals–a review. Anim. Genet..

[14] Demir E., Ceccobelli S., Bilginer U., Pasquini M., Attard G., Karsli T. (2022). Conservation and selection of genes related to environmental adaptation in native small ruminant breeds: A review. Ruminants.

[15] Saravanan K.A., Panigrahi M., Kumar H., Bhushan B. (2022). Advanced software programs for the analysis of genetic diversity in livestock genomics: A mini review. Biol. Rhythm Res..

[16] Bilginer U., Demir E., Karsli B.A., Doğru H., Kaya S., Meydan H., Yağcı S., Karacaören B., Karsli T. (2025). Unveiling genomic diversity and population structure in three Anatolian goats at genome-wide level. Small Rumin. Res..

[17] Vani A., Kumar A., Mahala S., Janga S.C., Chauhan A., Mehrotra A., De A.K., Sahu A.R., Ahmad S.F., Vempadapu V. (2024). Revelation of genetic diversity and genomic footprints of adaptation in Indian pig breeds. Gene.

[18] Karsli T. (2025). Genome-wide genetic characterization and selection signatures in Anatolian Merino sheep. Arch. Anim. Breed..

[19] Demir E., Karsli B.A., Özdemir D., Bilginer U., Doğru H., Kaya S., Atmaca V., Tufan N., Demir E., Karsli T. (2025). Genome-wide comparative analysis of variability and population structure between autochthonous Turkish chicken breeds and commercial hybrid lines. Poult. Sci..

[20] Vineeth M.R., Surya T., Sivalingam J., Kumar A., Niranjan S.K., Dixit S.P., Singh K., Tantia M.S., Gupta I.D. (2020). Genome-wide discovery of SNPs in candidate genes related to production and fertility traits in Sahiwal cattle. Trop. Anim. Health Prod..

[21] Tian D., Sun D., Ren Q., Zhang P., Zhang Z., Zhang W., Luo H., Li X., Han B., Liu D. (2023). Genome-wide identification of candidate copy number polymorphism genes associated with complex traits of Tibetan-sheep. Sci. Rep..

[22] Nayak S.S., Panigrahi M., Rajawat D., Sahoo S.P., Dutt T. (2025). Genomic scans for diversity and selection signatures in Indian Red Sindhi cattle. Mamm. Genome.

[23] Karsli B.A. (2024). Genetic diversity and population structure of four Anatolian sheep revealed by genome-wide ddRADseq data. Small Rumin. Res..

[24] Peterson B.K., Weber J.N., Kay E.H., Fisher H.S., Hoekstra H.E. (2012). Double digest RADseq: An inexpensive method for de novo SNP discovery and genotyping in model and non-model species. PLoS ONE.

[25] Rochette N.C., Rivera-Colón A.G., Catchen J.M. (2019). Stacks 2: Analytical methods for paired-end sequencing improve RADseq-based population genomics. Mol. Ecol..

[26] Chen S., Zhou Y., Chen Y., Gu J. (2018). fastp: An ultra-fast all-in-one FASTQ preprocessor. Bioinformatics.

[27] Langmead B., Salzberg S.L. (2012). Fast gapped-read alignment with Bowtie 2. Nat. Methods.

[28] Danecek P., Bonfield J.K., Liddle J., Marshall J., Ohan V., Pollard M.O., Whitwham A., Keane T., McCarthy S.A., Davies R.M. (2021). Twelve years of SAMtools and BCFtools. Gigascience.

[29] Chang C.C., Chow C.C., Tellier L.C., Vattikuti S., Purcell S.M., Lee J.J. (2015). Second-generation PLINK: Rising to the challenge of larger and richer datasets. Gigascience.

[30] Rambaut A. (2018). FigTree v.1.4.4. https://github.com/rambaut/figtree.

[31] Alexander D.H., Novembre J., Lange K. (2009). Fast model-based estimation of ancestry in unrelated individuals. Genome Res..

[32] Milanesi M., Capomaccio S., Vajana E., Bomba L., Garcia J.F., Ajmone-Marsan P., Colli L. (2017). BITE: An R package for biodiversity analyses. bioRxiv.

[33] Biscarini F., Cozzi P., Gaspa G., Marras G. (2019). Detect Runs of Homozygosity and Runs of Heterozygosity in Diploid Genomes. https://cran.r-project.org/web/packages/detectRUNS/vignettes/detectRUNS.vignette.html.

[34] Liu J., Shi L., Li Y., Chen L., Garrick D., Wang L., Zhao F. (2021). Estimates of genomic inbreeding and identification of candidate regions that differ between Chinese indigenous sheep breeds. J. Anim. Sci. Biotechnol..

[35] Gautier M., Klassmann A., Vitalis R. (2017). rehh 2.0: A reimplementation of the R package rehh to detect positive selection from haplotype structure. Mol. Ecol. Resour..

[36] Browning B.L., Tian X., Zhou Y., Browning S.R. (2021). Fast two-stage phasing of large-scale sequence data. Am. J. Hum. Genet..

[37] Voight B.F., Kudaravalli S., Wen X., Pritchard J.K. (2006). A Map of Recent Positive Selection in the Human Genome. PLoS Biol..

[38] Fonseca P.A., Suárez-Vega A., Marras G., Cánovas Á. (2020). GALLO: An R package for genomic annotation and integration of multiple data sources in livestock for positional candidate loci. Gigascience.

[39] Kominakis A., Tarsani E., Hager-Theodorides A.L., Mastranestasis I., Gkelia D., Hadjigeorgiou I. (2021). Genetic differentiation of mainland-island sheep of Greece: Implications for identifying candidate genes for long-term local adaptation. PLoS ONE.

[40] Wolf M.J., Yin T., Neumann G.B., Korkuć P., Brockmann G.A., König S., May K. (2021). Genome-wide association study using whole-genome sequence data for fertility, health indicator, and endoparasite infection traits in German black pied cattle. Genes.

[41] Campos B.M., do Carmo A.S., da Silva T.B.R., Verardo L.L., de Simoni Gouveia J.J., Malhado C.H.M., da Silva M.V.G.B., Carneiro P.L.S. (2017). Identification of artificial selection signatures in Caracu breed lines selected for milk production and meat production. Livest. Sci..

[42] Abousoliman I., Reyer H., Oster M., Murani E., Mohamed I., Wimmers K. (2021). Genome-wide analysis for early growth-related traits of the locally adapted Egyptian Barki sheep. Genes.

[43] Zhang L., Zhang S., Yuan M., Zhan F., Song M., Shang P., Yang F., Li X., Qiao R., Han X. (2023). Genome-wide association studies and runs of homozygosity to identify reproduction-related genes in Yorkshire pig population. Genes.

[44] Qiao L., Ma K., Yao Q., Zhang S., Pang Z., Wang W., Cai K., Liu W. (2025). Whole-Genome Sequencing of Dorper× Hu Hybrid Sheep for Screening Selection Signatures Associated with Litter Size. Animals.

[45] Ding W., Wu X., Bu Y., Zhang W., Wang Y., Ding Y., Zheng X., Zhang X., Yin Z. (2026). Runs of homozygosity reveal candidate genes for economic traits in Danish Large White pigs. Arch. Anim. Breed..

[46] Zhao F., Deng T., Shi L., Wang W., Zhang Q., Du L., Wang L. (2020). Genomic scan for selection signature reveals fat deposition in Chinese indigenous sheep with extreme tail types. Animals.

[47] Tao L., Wang X., Zhong Y., Liu Q., Xia Q., Chen S., He X., Di R., Chu M. (2021). Combined approaches identify known and novel genes associated with sheep litter size and non-seasonal breeding. Anim. Genet..

[48] Jin M., Wang H., Liu G., Lu J., Yuan Z., Li T., Liu E., Lu Z., Du L., Wei C. (2024). Whole-genome resequencing of Chinese indigenous sheep provides insight into the genetic basis underlying climate adaptation. Genet. Sel. Evol..

[49] Wu X., Pei J., Xiong L., Ge Q., Bao P., Liang C., Yan P., Guo X. (2025). Genome-wide scan for selection signatures reveals novel insights into the adaptive capacity characteristics in three Chinese cattle breeds. BMC Genom..

[50] Zhang H., Fu Y., Wang Y., Yin S., Jiang Y., Sun Y., Kang L. (2026). Genome-wide association study reveals candidate genes associated with key egg production traits in Langya chickens. Poult. Sci..

[51] Hamadalahmad A., Shariati M.M., Taheri S., Javadmanesh A. (2025). Identification of genes related to the trait of litter size in prolificacy breeds using Genome-wide Association Studies (GWAS). J. Rumin. Res..

[52] Selionova M., Aibazov M., Mamontova T., Malorodov V., Sermyagin A., Zinovyeva N., Easa A.A. (2022). Genome-wide association study of live body weight and body conformation traits in young Karachai goats. Small Rumin. Res..

[53] Abdulrahman K., Egor P.B., Fedor S.S. (2025). Weighted Single-Step Genome-Wide Association Study of Milk Production Traits in the Russian Black-and-White cattle. J. Biotechnol. Its App..

[54] Ospina A.T., Lewis R.M., Freking B.A., Burke J.M., Murphy T.W., Wilson C.S., Brito L.F. (2026). Genetic Parameters and Genome-Wide Association Studies for Fertility and Reproduction Traits in US Katahdin Sheep Based on the Single-Step GBLUP Methodology. J. Anim. Breed. Genet..

[55] Li Y., Chen Z., Fang Y., Cao C., Zhang Z., Pan Y., Wang Q. (2022). Runs of homozygosity revealed reproductive traits of Hu sheep. Genes.

[56] Tsartsianidou V., Otapasidis A., Papakostas S., Karaiskou N., Vouraki S., Triantafyllidis A. (2024). Genome-Wide patterns of homozygosity and heterozygosity and candidate genes in Greek insular and Mainland native goats. Genes.

[57] Pegolo S., Bisutti V., Mota L.F.M., Cecchinato A., Amalfitano N., Dettori M.L., Pazzola M., Vacca G.M., Bittante G. (2025). Genome-wide landscape of genetic diversity, runs of homozygosity, and runs of heterozygosity in five Alpine and Mediterranean goat breeds. J. Anim. Sci. Biotechnol..

[58] Onzima R.B., Upadhyay M.R., Doekes H.P., Brito L.F., Bosse M., Kanis E., Groenen M.A., Crooijmans R.P. (2018). Genome-wide characterization of selection signatures and runs of homozygosity in Ugandan goat breeds. Front. Genet..

[59] Fernandes Júnior G.A., da Silva M.V.G.B., Lobo A.M.B.O., Panetto J.C.D.C., de Moraes Silva K., Paiva S.R., Facó O. (2025). Genome scan for runs of homozygosity in Saanen goats selected under tropical environments reveals candidate genes for adaptive and production traits. Trop. Anim. Health Prod..

[60] Deniskova T.E., Dotsev A.V., Koshkina O.A., Solovieva A.D., Churbakova N.A., Petrov S.N., Frolov A.N., Platonov S.A., Abdelmanova A.S., Vladimirov M.A. (2025). Examination of runs of homozygosity distribution patterns and relevant candidate genes of potential economic interest in Russian goat breeds using whole-genome sequencing. Genes.

[61] Colombi D., Perini F., Bettini S., Mastrangelo S., Abeni F., Conte G., Marletta D., Cassandro M., Bernabucci U., Ciampolini R. (2024). Genomic responses to climatic challenges in beef cattle: A review. Anim. Genet..

[62] Peng W., Zhang Y., Gao L., Wang S., Liu M., Sun E., Lu K., Zhang Y., Li B., Li G. (2025). Investigation of selection signatures of dairy goats using whole-genome sequencing data. BMC Genom..

[63] Su R., Zhou H., Yang W., Moqir S., Ritu X., Liu L., Shi Y., Dong A., Bayier M., Letu Y. (2024). Near telomere-to-telomere genome assembly of Mongolian cattle: Implications for population genetic variation and beef quality. Gigascience.

[64] Kanlisi R.A., Amuzu-Aweh E.N., Naazie A., Otsyina H.R., Kelly T.R., Gallardo R.A., Lamont S.J., Zhou H., Dekkers J., Kayang B.B. (2024). Genetic architecture of body weight, carcass, and internal organs traits of Ghanaian local chickens. Front. Genet..

[65] Edea Z., Jeoung Y.H., Shin S.S., Ku J., Seo S., Kim I.H., Kim S.W., Kim K.S. (2018). Genome–wide association study of carcass weight in commercial Hanwoo cattle. Asian-Australas. J. Anim. Sci..

[66] Ding R., Qiu Y., Zhuang Z., Ruan D., Wu J., Zhou S., Ye J., Cao L., Hong L., Xu Z. (2021). Genome-wide association studies reveals polygenic genetic architecture of litter traits in Duroc pigs. Theriogenology.

[67] Ramos Z., Garrick D.J., Blair H.T., Vera B., Ciappesoni G., Kenyon P.R. (2023). Genomic regions associated with wool, growth and reproduction traits in Uruguayan Merino sheep. Genes.

[68] Vani S., Balasubramanyam D., Karthickeyan S.M.K., Tirumurugan K.G., Gopinathan A., Hepsibha P., Madhuri B.J. (2026). Genomewide signatures revealing new evidence for selection of important traits in indigenous cattle breeds of Tamil Nadu, India. J. Genet..

[69] Kudinov A.A., Dementieva N.V., Mitrofanova O.V., Stanishevskaya O.I., Fedorova E.S., Larkina T.A., Mishina A.O., Plemyashov K.V., Griffin D.K., Romanov M.N. (2019). Genome-wide association studies targeting the yield of extraembryonic fluid and production traits in Russian White chickens. BMC Genom..

[70] Zhao Y., He S., Huang J., Liu M. (2023). Genome-wide association analysis of muscle pH in Texel Sheep× Altay Sheep F2 resource population. Animals.

[71] Chernet T.F., Tarekegn G.M., Ally O.M., Meseret S., Mrode R., Negussie E., Bedada Z.E., Gebreyohanes G., Ekine-Dzivenu C., Tera A. (2025). Single-step genome-wide association study of milk somatic cell scores across multi-cattle breeds in Ethiopia. Anim. Biotechnol..

[72] Shen Z., Yang L., Xue Y., Chang X., Shen J., Sun W., Zeng Y., Meng J., Yao X. (2026). Machine Learning-Based Genome-Wide Association Study Reveals Genetic Loci Associated with Body Measurement Traits in Yili Horses. Animals.

[73] Pedrosa V.B., Schenkel F.S., Chen S.Y., Oliveira H.R., Casey T.M., Melka M.G., Brito L.F. (2021). Genomewide association analyses of lactation persistency and milk production traits in Holstein cattle based on imputed whole-genome sequence data. Genes.

[74] Saravanan K.A., Panigrahi M., Kumar H., Parida S., Bhushan B., Gaur G.K., Dutt T., Mishra B.P., Singh R.K. (2021). Genomic scans for selection signatures revealed candidate genes for adaptation and production traits in a variety of cattle breeds. Genomics.

[75] Hollmann A.K., Bleyer M., Tipold A., Neßler J.N., Wemheuer W.E., Schütz E., Brenig B. (2017). A genome-wide association study reveals a locus for bilateral iridal hypopigmentation in Holstein Friesian cattle. BMC Genet..

[76] Mastrangelo S., Cendron F., Sottile G., Niero G., Portolano B., Biscarini F., Cassandro M. (2020). Genome-wide analyses identifies known and new markers responsible of chicken plumage color. Animals.

[77] Melo-Rojas C., Bravo-Matheus P.W., Amaht Araoz C., Zapata-Coacalla C. (2023). Identification of polymorphisms in TYRP1, DCT and RAB38 genes and their association with coat color in alpacas. Front. Anim. Sci..

[78] Bayraktar M., Durmuş M., Uyguner Ö., Turan M., Özcan B.D., Koluman N. (2026). Comprehensive analysis of population structure and genomic diversity in fifteen Anatolian sheep breeds. BMC Vet. Res..

[79] Dzomba E.F., Chimonyo M., Pierneef R., Muchadeyi F.C. (2021). Runs of homozygosity analysis of South African sheep breeds from various production systems investigated using OvineSNP50k data. BMC Genom..

[80] Yilmaz O., Cemal I., Karaca O. (2014). Genetic diversity in nine native Turkish sheep breeds based on microsatellite analysis. Anim. Genet..

[81] Ozmen O., Kul S., Gok T. (2020). Determination of genetic diversity of the Akkaraman sheep breed from Turkey. Small Rumin. Res..

[82] Ceballos F.C., Joshi P.K., Clark D.W., Ramsay M., Wilson J.F. (2018). Runs of homozygosity: Windows into population history and trait architecture. Nat. Rev. Genet..

[83] Li R., Zhao Y., Liang B., Pu Y., Jiang L., Ma Y. (2023). Genome-wide signal selection analysis revealing genes potentially related to sheep-milk-production traits. Animals.

[84] Astuti P.K., Mizeranschi A.E., Herdis H., Sitaresmi P.I., Ibrahim A., Prastowo S., Widyas N., Nugroho T., Kusza S. (2026). Putative Genomic Signatures of Local Adaptation in Five Local Indonesian Sheep Reveal Selection on Immunity, Reproduction, and Production Traits. Ecol. Evol..

[85] Quazi F., Molday R.S. (2011). Lipid transport by mammalian ABC proteins. Essays Biochem..

[86] Di Zazzo E., De Rosa C., Abbondanza C., Moncharmont B. (2013). PRDM proteins: Molecular mechanisms in signal transduction and transcriptional regulation. Biology.

[87] Singh N., Patra S. (2014). Phosphodiesterase 9: Insights from protein structure and role in therapeutics. Life Sci..

[88] Casarini L., Paradiso E., Lazzaretti C., D’Alessandro S., Roy N., Mascolo E., Zareba K., García-Gasca A., Simoni M. (2022). Regulation of antral follicular growth by an interplay between gonadotropins and their receptors. J. Assist. Reprod. Genet..

[89] Schmitz G., Langmann T., Heimerl S. (2001). Role of ABCG1 and other ABCG family members in lipid metabolism. J. Lipid Res..

[90] Perini F., Wu Z., Cartoni Mancinelli A., Soglia D., Schiavone A., Mattioli S., Mugnai C., Castellini C., Smith J., Lasagna E. (2023). RNAseq reveals modulation of genes involved in fatty acid biosynthesis in chicken liver according to genetic background, sex, and diet. Anim. Genet..

[91] Li L., Yang Y., Yang G., Lu C., Yang M., Liu H., Zong H. (2011). The role of JAZF1 on lipid metabolism and related genes in vitro. Metabolism.

[92] Huang H., Lee D.H., Zabolotny J.M., Kim Y.B. (2013). Metabolic actions of Rho-kinase in periphery and brain. Trends Endocrinol. Metab..

[93] Porto-Neto L.R., Reverter A., Prayaga K.C., Chan E.K., Johnston D.J., Hawken R.J., Fordyce G., Garcia J.F., Sonstegard T.S., Bolormaa S. (2013). The genetic architecture of climatic adaptation of tropical cattle. PLoS ONE.

[94] Demir E., Bilginer U., Doğru H., Karacaören B., Meydan H., Çiftçi Z., Yağci S., Kaya S., Karsli T. (2026). Candidate genes related to growth and milk production in three Anatolian goats revealed by GWAS. Mamm. Genome.

[95] Rabiu L., Zhang P., Afolabi L.O., Saliu M.A., Dabai S.M., Suleiman R.B., Gidado K.I., Ige M.A., Ibrahim A., Zhang G. (2024). Immunological dynamics in MASH: From landscape analysis to therapeutic intervention. J. Gastroenterol..

[96] Li F., Cui X., Yang Q., Nie Y., Wang J., Sun S. (2025). The research progress of LACC1. Front. Immunol..

[97] Wang Y., Niu Z., Zeng Z., Jiang Y., Jiang Y., Ding Y., Tang S., Shi H., Ding X. (2020). Using high-density SNP array to reveal selection signatures related to prolificacy in Chinese and Kazakhstan sheep breeds. Animals.

[98] Sánchez-Ramos R., Trujano-Chavez M.Z., Gallegos-Sánchez J., Becerril-Pérez C.M., Cadena-Villegas S., Cortez-Romero C. (2023). Detection of candidate genes associated with fecundity through genome-wide selection signatures of Katahdin ewes. Animals.

